# International Humanitarian Law: what does it say on protection of medical facilities?

**DOI:** 10.1093/eurpub/ckab168

**Published:** 2021-09-25

**Authors:** Ove Bring

**Affiliations:** 1 Stockholm University, Stockholm, Sweden; 2 Former Member of the Permanent Court of Arbitration, the Hague, Netherlands; 3 Former President of the Swedish Branch of the International Law Association, Sweden

With experience from research and policy on international humanitarian law, I will here give some short comments from an international law point of view.

With regard to the recurring hostilities linked to Gaza, it is well known that Israeli forces have in some cases attacked medical facilities and thus committed grave breaches of international humanitarian law (IHL). On the other hand, it cannot be excluded that the common Israeli argument that medical facilities have been shielding military resources has sometimes been correct. Nevertheless, it is clear that Israel has not always taken feasible precautions in order not to harm civilians, including medical personnel. The obligation of feasible precautions is a cornerstone of IHL.

At the same time, the protection to which medical units are entitled will cease if they are used for military purposes harmful to the enemy. The parties to the conflict shall not use civilians or medical units in order to attempt to shield military objectives from attacks. Nevertheless, the protection of medical units shall cease only after a fair warning has been given, setting a time limit, and after such warning has remained unheeded.

It is a tragedy that these rules of international humanitarian law have often been violated by parties to armed conflicts, especially in the hostilities between Hamas and Israel.

